# Identification of Behavioral, Clinical, and Psychological Antecedents of Acute Stimulant Poisoning: Development and Implementation of a Mixed Methods Psychological Autopsy Study

**DOI:** 10.2196/64873

**Published:** 2025-01-13

**Authors:** Marley Antolin Muñiz, Vanessa M McMahan, Xochitl Luna Marti, Sarah Brennan, Sophia Tavasieff, Luke N Rodda, James Knoll, Phillip O Coffin

**Affiliations:** 1 Center on Substance Use and Health, San Francisco Department of Public Health, San Francisco, CA, United States; 2 Forensic Laboratory Division, Office of the Chief Medical Examiner, San Francisco, CA, United States; 3 Department of Medicine, University of California, San Francisco, San Francisco, CA, United States; 4 Department of Psychiatry, SUNY Upstate Medical University, Syracuse, NY, United States

**Keywords:** psychological autopsy, acute stimulant poisoning, overdose, cocaine, methamphetamine, fentanyl

## Abstract

**Background:**

Despite increasing fatal stimulant poisoning in the United States, little is understood about the mechanism of death. The psychological autopsy (PA) has long been used to distinguish the manner of death in equivocal cases, including opioid overdose, but has not been used to explicitly explore stimulant mortality.

**Objective:**

We aimed to develop and implement a large PA study to identify antecedents of fatal stimulant poisoning, seeking to maximize data gathering and ethical interactions during the collateral interviews.

**Methods:**

We ascertained death records from the California Electronic Death Reporting System (CA-EDRS) and the San Francisco Office of the County Medical Examiner (OCME) from June 2022 through December 2023. We selected deaths determined to be due to acute poisoning from cocaine or methamphetamine, which occurred 3‐12 months prior and were not attributed to suicide or homicide. We identified 31 stimulant-fentanyl and 70 stimulant-no-opioid decedents. We sought 2 informants for each decedent, who were able to describe the decedent across their life course. Informants were at least 18 years of age, communicated with the decedent within the year before death, and were aware that the decedent had been using substances during that year. Upon completion of at least one informant interview conducted by staff with bachelor’s or master’s degrees, we collected OCME, medical record, and substance use disorder treatment data for the decedent. Planned analyses include least absolute shrinkage and selection operator regressions of quantitative data and thematic analyses of qualitative data.

**Results:**

We identified and interviewed at least one informant (N=141) for each decedent (N=101). Based on feedback during recruitment, we adapted language to improve rapport, including changing the term “accidental death” to “premature death,” offering condolences, and providing content warnings. As expected, family members were able to provide more data about the decedent’s childhood and adolescence, and nonfamily informants provided more data regarding events proximal to death. We found that the interviews were stressful for both the interviewee and interviewer, especially when participants thought the study was intrusive or experienced significant grief during the interviews.

**Conclusions:**

In developing and implementing PA research on fatal stimulant poisoning, we noted the importance of recruitment language regarding cause of death and condolences with collateral informants. Compassion and respect were critical to facilitate the interview process and maintain an ethical framework. We discuss several barriers to success and lessons learned while conducting PA interviews, as well as recommendations for future PA studies.

## Introduction

Deaths attributed to acute stimulant poisoning have been increasing in the United States [1]. From 2015 to 2022, poisoning deaths in the United States attributed to cocaine or methamphetamine increased 4- and 6-fold, respectively [2]. This increase in mortality, largely driven by deaths also involving fentanyl, a potent synthetic opioid, has been referred to as the “fourth wave” of the overdose crisis [3-6]. While the increase in deaths involving stimulants and fentanyl may be primarily due to the toxic effects of fentanyl, the presence of stimulants likely reflects rising polysubstance and, in some cases, unintentional fentanyl use [4,5,7].

Fatal opioid overdose has a well-defined mechanism—respiratory depression eventually leading to cardiac arrest and death—and decades of research exploring the nature of and antecedents to death. However, this framework and associated research are absent for fatal stimulant poisoning, leading investigators and public health providers to assume stimulant poisoning deaths are similar to opioid overdose deaths and a result of an “overdose.” This has led to efforts to identify prevention and treatment strategies, like bystander interventions, reversal agents, and strategies to prevent an acute overdose, akin to strategies used for opioid overdose. However, deaths attributed to stimulant poisoning, which are attributed to cardiac or cerebrovascular events far more often than for opioid overdose deaths, may actually be due to chronic disease rather than a single event of substance use [8,9]. Thus, prevention efforts focused on bystander interventions may not be effective to prevent stimulant poisoning deaths, but instead longer-term interventions, such as screening, prevention, and treatment of chronic diseases, may be appropriate [10].

In the Leveraging Psychological Autopsies to Accelerate Research into Stimulant Overdose Mortality study, we aimed to identify the clinical, behavioral, and psychological antecedents of fatal stimulant poisoning. We selected the psychological autopsy (PA) [11,12], which has been used for suicide [12-15] and opioid overdose research [16,17], because this exploratory approach identified concepts decades ago that still drive opioid overdose prevention today [18]. We hypothesized that fatal stimulant poisoning likely has individual, social, and environmental contributors, and thus employed a socioecological conceptual framework [19]. To identify multilevel antecedents of fatal stimulant poisoning, we conducted PAs involving detailed interviews with close contacts of decedents (collateral informants), analyzed alongside medical examiner (case narrative, autopsy, and toxicology reports) and medical record data [20]. We describe the adaptation of PAs to study fatal stimulant poisoning and lessons learned.

## Methods

### Ethical Considerations

We obtained approval for this study from the University of California San Francisco, Human Research Protection Program (#21‐35305). To use informant information in death records from the California Electronic Death Reporting System (CA-EDRS), we also received approval from the State of California Health and Human Services Agency Committee for the Protection of Human Subjects (#2022‐091).

### Decedent Case Selection

We reviewed records from the CA-EDRS and the Office of the County Medical Examiner (OCME) of San Francisco for eligible cases from June 2022 through December 2023. We selected closed cases of deaths determined to be due to acute poisoning from cocaine or methamphetamine. Suicides and homicides were excluded because the mechanism and antecedents to death would likely differ from accidental, natural, or undetermined deaths [21]. We sought 30 stimulant-fentanyl decedents (15 involving cocaine and 15 involving methamphetamine) and 70 stimulant-no-opioid decedents (35 involving cocaine and 35 involving methamphetamine). We suspected that stimulant-fentanyl deaths would be similar to opioid deaths, which are well understood. However, little is known about stimulant deaths not involving opioids, thus we targeted a larger sample of that group [9]. Decedents without a known name or informant were excluded. Interviews were conducted 3‐12 months after decedent death to minimize both acute bereavement and forgetfulness among informants [22,23]. Decedents were considered “enrolled” in the study when at least one informant interview was completed, at which time OCME, medical record, and substance use treatment data were collected.

### Informant Selection

We sought 2 informants for each decedent, who ideally could describe the decedent across the life course (eg, childhood experiences and behaviors proximal to death). Informants had to be at least 18 years of age, have communicated with the decedent within the year before death, be aware that the decedent was using substances during that year, and be able to provide informed consent in English. We identified potential informants using CA-EDRS and OCME data, medical record review, flyers, and word of mouth. We conducted outreach at harm reduction programs and public housing settings where decedents had resided. If a mailing address was available for a potential informant, we mailed a recruitment letter that included condolences, the purpose of the study, details of participation, and the option to opt out of additional contact from the study team (Multimedia Appendix 1). We then waited 1 week before reaching out by phone if a phone number was available. If only a phone number was available, we reached out immediately with up to 3 calls, typically separated by at least 1 week.

### Interview Procedures

Bachelor’s and master’s-level staff conducted the interviews after receiving 2 hours of training by a psychiatrist experienced in PAs. All informants provided informed consent through review of an information sheet. Semistructured, mixed methods interviews lasting 1‐2 hours were then conducted over telephone, Zoom, or in person. The interview included 14 thematic sections involving sociodemographic characteristics, neighborhood and housing, adverse childhood experiences, emotional and psychological health, substance use, and social environment (Multimedia Appendix 1). Each section began with quantitative questions, including validated scales, followed by qualitative questions to add nuance and to contextualize quantitative data. Quantitative interview responses were entered into University of California San Francisco’s REDCap (Research Electronic Data Capture) [24,25] in real time by the interviewer. Interviews were audio-recorded and transcribed verbatim via the Rev platform. Informants were compensated US $50 and offered an additional US $10 for any referral that resulted in a completed informant interview. After the interview, informants were offered bereavement resources and additional information about the cause of death as appropriate. Because some informants had superior knowledge about different aspects of a decedent’s life, the interviewer scored the reliability of the responses to each of the survey sections as highly unreliable, somewhat unreliable, neutral, somewhat reliable, or highly reliable.

### Additional Data Sources

Upon completion of at least one informant interview, we collected additional OCME, medical record, and substance use disorder treatment data. We waited until the informant interview was completed to ensure we only collected these data for enrolled decedents. The data abstraction form was developed based on prior research the team had conducted [26], the Centers for Disease Control and Prevention (CDC) State Unintentional Drug Overdose Reporting System (SUDORS) [27], and a preliminary review of 15 decedents. OCME data included the case narrative, toxicology, and autopsy reports. Medical record data included regularity of medical care (eg, established primary care, urgent care only, emergency or hospital care only); recent emergency or inpatient care; latest electrocardiogram and echocardiogram results; latest troponin, brain natriuretic peptide, and urine drug screen results; any evidence of opioid, cocaine, or methamphetamine use; all problem list diagnoses; and all active medications at the time of death.

### Analytic Plan

We plan to conduct descriptive statistics of quantitative data, with a focus on a priori variables of interest, including cardiac health, social isolation, fentanyl exposure, suicidality, and resilience. We will then conduct least absolute shrinkage and selection operator (LASSO) regression analyses [28] to identify variables related to fatal stimulant poisoning and analyze results by fentanyl involvement and by cocaine compared to methamphetamine as the causal stimulant. We will also conduct targeted, hypothesis-driven regression analyses by fentanyl involvement exploring cardiac health and opioid use history. Qualitative data will be analyzed by study team members using ATLAS.ti 23.2.2 with an a priori codebook refined during the consensus-driven coding process. Qualitative analyses will focus on salient and recurrent themes and describe theme co-occurrence.

## Results

**Figure 1.**
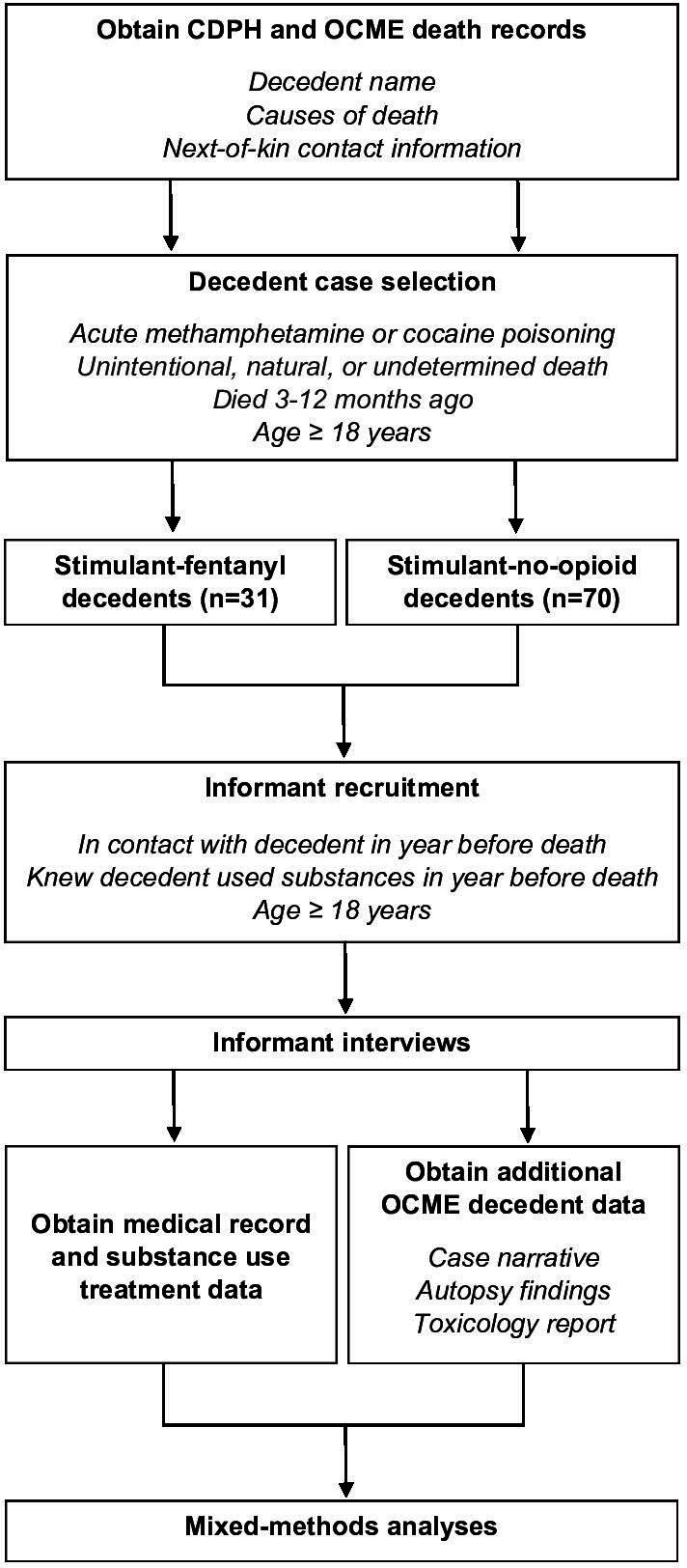
Procedural flow for a mixed methods psychological autopsy study of decedents of fatal stimulant poisoning in San Francisco, CA, from June 2022 to December 2023. CDPH: California Department of Public Health; OCME: Office of the County Medical Examiner.

We adapted the PA approach to conduct a large study of the antecedents of fatal stimulant poisoning (Figure 1). We identified and interviewed at least one informant for each decedent. During this period, we reviewed 177 potential stimulant-fentanyl cases prior to enrolling 31 decedents (18% of potential decedents; an extra decedent was erroneously enrolled) and 187 potential stimulant-no-opioid cases prior to enrolling 70 decedents (37% of potential decedents). For 19 (5%) cases, there were no informants available. We contacted 725 potential informants for a total of 364 decedents; 410 (57%) did not complete an interview or follow-up with the study team, 112 (15%) were not interested in participating, 62 (9%) were ineligible, and 141 (19%) completed an interview (Table 1).

Recruitment phone calls varied in length, usually lasting 15 minutes, or as long as 40 minutes when a potential informant was in emotional distress. Calls that were longer typically seemed welcome by potential participants, during which they discussed the lives of the decedents and their own feelings of grief. In some calls, potential participants also discussed frustration with the unexpected contact.

Most potential informants were family members (n=409, 56%), including 133 (18%) parents, 80 (11%) children, and 196 (27%) siblings or other family. We also contacted friends (n=79, 11%), spouses or partners (n=43, 6%), and service providers (n=49, 7%). Many decedents had lived in supportive housing settings (eg, single residence occupancy hotels); thus, 77 (11%) potential informants were building staff. Informants who completed interviews had a similar distribution (Table 2).

The majority of stimulant-involved deaths in San Francisco were also attributed to fentanyl [29], thus we rapidly enrolled stimulant-fentanyl decedents. We then shifted our focus to enrolling stimulant-no-opioid decedents, at which point 2 potential informants reached out to us with concerns about the study procedures; both were concerned with the unexpected outreach, the lack of condolences offered in our recruitment materials, and the phrase “accidental death” used to describe the purpose of the study. In dialogue with these potential informants, it became clear that they considered “accidental death” to mean “overdose,” and they did not feel that their loved one had died from an “overdose.”

We subsequently revised the recruitment letter and our telephone script to begin with content warnings about the sensitivity of the topic we would be discussing, refer to “premature death,” instead of “accidental death,” offer condolences, and allow for more check-ins. We also made the introduction of our recruitment materials intentionally vague and did not discuss the study’s focus of fatal stimulant poisoning until the individual expressed comfort with talking with us, we suggested they speak with us from a private location, and we confirmed their eligibility (Multimedia Appendix 1). These changes were made to promote comfort and engagement among potential informants during recruitment and to limit disclosure of the involvement of stimulants in death to individuals without prior knowledge of the decedent’s substance use. After making these changes, we completed all remaining interviews with only one potential informant voicing concern about the purpose of the study. We speculate that the potential informant had reviewed our center’s website, which was included on the letterhead of the recruitment letter, providing them with discomforting details about the study.

In addition to the modified recruitment language, we made several other modifications to the study during implementation. Communicating with potential informants proved challenging via telephone calls only, so we modified the study to allow for text messaging with informants. We only corresponded via text message when contact had already been established with the potential informant. After recruitment commenced, it became clear that it would be challenging to interview 2 informants for each decedent, so we modified the study to allow for only one informant if we could not reach a second informant. We also allowed for more than 2 informants in the event one informant who was interviewed was not knowledgeable about the decedent’s life. We modified our inclusion criteria to allow for the enrollment of decedents who died from both methamphetamine and cocaine toxicity, since we did not think we would lose scientific information by their inclusion, and, depending on the number enrolled, we might be able to identify unique characteristics of this group. Finally, during the study we added additional recruitment strategies, including snowball sampling (ie, compensating a participant who referred another participant an additional US $10), contacting participants after the interview who had consented to being contacted for recruitment purposes, and posting flyers in settings where we could reach potential informants (eg, syringe services programs, public housing, etc).

Interviews lasted 1-2 hours. Interviewers coded the reliability of informants for each section of questions in the interview. In general, family members had superior information about childhood experiences and less reliable information about recent substance use. Staff experienced stress in conducting the interviews, particularly when potential informants felt that recruitment efforts were intrusive or when enrolled informants experienced significant grief during interviews. Staff were not specifically trained in psychology or bereavement counseling and at times were overwhelmed by the grief among informants. To alleviate some of the emotional burden on the study team, limits were set around interview capacity, with interviewers performing no more than 1 interview daily and a maximum of 3 interviews per week. Weekly team calls included conversations about recent informant interactions and upcoming capacity for recruitment calls and interviews. Staff could also access biweekly debrief sessions led by a clinical psychologist on staff, addressing topics such as the varying signs of grief and how those signs may show up in outreach and interviews. Interviewers and recruiters were trained in motivational interviewing techniques to provide staff with tools like reflective listening to express empathy during outreach and interviews.

**Table 1.** The number of potential informants for eligible decedents and the recruitment status of potential informants contacted for a mixed methods psychological autopsy study of decedents of acute stimulant poisoning in San Francisco, CA, from June 2022 to December 2023.

	n	%
**Eligible decedents (N=364)^a^**		
No informants	19	5
At least one potential informant, not enrolled	244	67
At least one potential informant, enrolled	101	28
**Potential informants contacted (N=725)**		
Ineligible^b^	62	9
Refused participation	112	15
Contacted but no interview completed or did not follow-up^c^	410	57
Completed interview	141	19

a Closed cases due to cocaine or methamphetamine acute poisoning with and without fentanyl involvement. Deaths involving other opioids were excluded. Once 31 fentanyl-involved decedents were enrolled, eligibility was restricted to stimulant poisoning without any opioid involvement.

b Potential informants were ineligible if they were <18 years old, had not been in contact with the decedent in the year prior to death, did not know the decedent used substances in the year before death, were not able to provide informed consent in English, or the decedent had not died 3‐12 months before recruitment.

c Includes potential informants who were sent a letter or called but never responded, as well as those who were successfully contacted but never followed up to complete an interview. Tracking database did not allow for further disaggregation.

**Table 2.** The relationships to decedents of potential informants who were contacted and interviewed for a mixed methods psychological autopsy study of decedents of fatal stimulant poisoning in San Francisco, CA, from June 2022 to December 2023.

Relationship of informant to decedent	Contacted (N=725)		Interviewed (N=141)	
	n	%	n	%
Parent	133	18	18	13
Spouse or partner	43	6	8	6
Child	80	11	17	12
Friend	79	11	30	21
Sibling or other family member	196	27	44	31
Service provider (eg, clinician, case manager)	49	7	11	8
Building staff	77	11	4	3
Other	68	9	9	6

## Discussion

We successfully adopted the PA methodology for a large investigation of antecedents to fatal stimulant poisoning. We enrolled at least one informant for 101 decedents. We ascertained OCME and medical record data for all enrolled decedents. We identified challenges including recruiting informants, adapting our recruitment process to address informant grief, and ensuring staff were prepared to manage emotionally challenging interactions.

Enrolling informants was a substantial challenge of the project. We sought 2 informants per decedent; however, a high proportion of our decedents were markedly socially isolated. Nineteen of the decedents we initially identified were either unidentifiable (ie, “John Doe”) or had no contacts listed in their death or medical records. Among those who had any contacts, most had very few and often only family members, limiting our ability to obtain detailed information related to substance use. Few decedents had multiple contacts listed who knew the decedent proximal to death (eg, current friends, partners). We attempted to overcome these challenges by asking informants for referrals of others who knew the decedent and may be interested in participating in an interview, conducting direct outreach to public housing, posting flyers with our contact information, contacting health care providers for recommendations, and using personal connections research staff had with service programs in the community. We also altered the protocol to allow for a single informant when 2 could not be identified; as social isolation was a frequent antecedent to death, we felt this adjustment would help to reduce bias by including more socially isolated decedents. Nonetheless, a significant proportion of decedents could not be enrolled due to an inability to reach any eligible informants.

Because stimulant-no-opioid cases were far less common than stimulant-fentanyl cases, we devoted substantially more effort to locating informants and ended up enrolling a higher proportion of potential stimulant-no-opioid decedents (37%) compared to potential stimulant-fentanyl decedents (18%). Many potential informants were ineligible due to our requirement that the informant be aware of the decedent’s substance use, as well as speak sufficient English to provide informed consent. The dearth of informants for many decedents was compounded by ethical limitations in outreach efforts. Our human subjects committee required a letter be sent to potential informants with a mailing address but allowed direct phone contact for those without a mailing address.

Some informants experienced significant emotional distress during interviews and outreach. Potential informants received “cold calls” from study staff, rather than a warm handoff from a known provider, and did not directly consent to initial contact from our research team. This element of surprise and the sensitive nature of the call left some potential informants feeling the outreach was intrusive. However, for others who spoke at length during the recruitment calls, it seemed like a welcome opportunity to talk about the decedent. While a warm handoff from known clinical providers or the medical examiner’s office may be preferred for initial outreach, this may not be feasible for larger PA studies or non-research “death review” projects attempting to track and improve our understanding of drug poisoning deaths.

During the study, we adapted recruitment materials and processes to respond to concerns from potential informants, including more sympathetic language, frequent check-ins, and a content warning. We only noted the role of stimulants in the decedent’s death after confirming that the informant knew about the decedent’s substance use, and we replaced the phrase “accidental death” with “premature death.” These later changes were consistent with preliminary findings from early reviews of transcripts, as informants often did not perceive stimulant poisoning deaths to be “overdose” deaths but instead natural or medical deaths. Most potential informants reacted well to the improved script and recruitment approaches. As in other PA studies [30,31], many informants had a positive experience participating in the study and expressed gratitude to the study team for conducting the research, showing interest in the decedent’s life, and providing an opportunity for them to speak about the decedent.

During interviews, we checked in frequently, offered breaks, asked informants how the experience was for them, and if they would like any additional resources. Interviewers also offered bereavement resources consisting of a list of local and national bereavement meetings or websites. The content of recruitment calls and informant interviews was often emotionally draining for staff as well, which was expected given the topics addressed with close contacts of decedents [11]. Additional strategies to address grief among informants may be beneficial, such as postinterview check-ins with informants to offer additional resources [30]. Future PA studies may also benefit from employing study staff with a background in psychiatric work, experience with bereavement, experience with death investigations and next-of-kin notifications, or experience working with populations who use substances [16].

Despite these challenges, we were able to effectively use PAs to conduct a large study of fatal stimulant poisoning using data from multiple independent sources. The marked social isolation of people who use stimulants and the sensitive nature of interviews were significant challenges, some of which were distinct from studies of opioid overdose. The PA requires that informants voluntarily discuss their memories and insights about an extremely distressing event [31]. Thus, future developments in PA research should focus on informant and interviewer well-being. This could include adapting recruitment language to the specific population being studied, including the use of terms such as “premature death” rather than any specific mechanism of death; offering condolences, content warnings, and check-ins as needed; seeking out staff with experience in psychology, bereavement, and substance use; incorporating postinterview check-ins with informants; and using warm handoffs of informants when feasible.

## Supplementary material

10.2196/64873 Multimedia Appendix 1 No image included. Copies of outreach letter, REDCap (Research Electronic Data Capture) survey, and telephone recruitment script.

## Abbreviations

CA-EDRS: California Electronic Death Reporting System
CDC: Centers for Disease Control and Prevention
OCME: Office of the County Medical Examiner
PA: psychological autopsy
REDCap: Research Electronic Data Capture
SUDORS: State Unintentional Drug Overdose Reporting System
