# Supplementary material for: Identification of Behavioral, Clinical, and Psychological Antecedents of Acute Stimulant Poisoning: Development and Implementation of a Mixed Methods Psychological Autopsy Study
Source: JMIR Form Res. 2025 Jan 13;9:e64873. doi: 10.2196/64873 (PMC11745507; doi:10.2196/64873)
Supplement: Multimedia Appendix 1 [file formative-v9-e64873-s001.docx]

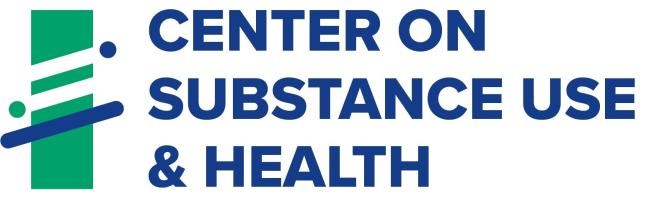


**www.**

**csuhsf.org**

**25**

**Van Ness Ave, Suite**

**500**

**San Francisco, CA 94102**

Dear ____________,

The content below discusses the death of ___________ (decedent’s name), who may have been very close to you. Please only proceed if comfortable doing so.

I hope this letter finds you well. My name is _______________ (staff member’s name) and I am reaching out on behalf of the San Francisco Department of Public Health. We are conducting a research study to learn more about how to prevent premature deaths. We are reaching out to you because you knew _______________ (decedent’s name). You may be aware that

_______________ (decedent’s name) passed away ___ months ago, and you were identified as a person who may have been close to them. I would like to offer my deepest condolences for your loss. I understand you may be in a time of grief and this topic may be sensitive and difficult to discuss, please only proceed if comfortable.

If you are comfortable participating, we would like to ask you to share your experience with ______________ (decedent’s name). Your story holds a great deal of value that could help inform future interventions that may prevent premature deaths. If you are eligible and interested in participating, we would offer you a safe space to have a conversation with one of our study team members, during which we would ask you details about _______________ (decedent’s name). We could talk with you either at our office, which is located at 25 Van Ness Avenue in San Francisco, or via Zoom or phone. The conversation would last approximately 1-2 hours. In exchange for your time, a $50 stipend is provided to eligible participants for completing the interview. Please know that while we would greatly appreciate your input, your participation is entirely voluntary, and you may decline participation at any time.

If you are interested, or would like more information about the study, please respond to this letter via email at _________________ or call me, at _______________ (staff member’s phone number). If you are not interested in participating but know someone else who knew

_______________ (decedent’s name) well who might be interested, they can contact us at the email address or phone number below, or you could send us their contact information.

If we don’t hear from you, a member of our staff may reach out to you via phone call in the next week. If you do not want us to contact you, you can opt-out by calling us at _______________.

With gratitude, _______________ (staff member’s signature and contact information)

The Center on Substance Use and Health is based out of the San Francisco Department of Public Health 1

**Recruitment Phone Script**

Hi ____, is this ________ [informant’s name]? Hi, _______[informant’s name], how are you doing? My name is ______ [recruiter’s name] and I’m calling regarding a research study at the San Francisco Department of Public Health. Do you have a few minutes to chat?

*[If no:]*

Ask if there is another time that they would prefer to be contacted at/that may be better for them.

*[If yes:]*

Before we begin, I want to let you know that this study does cover sensitive topics and may bring up difficult memories for you. Specifically, we would be talking about your _______ (relationship) by the name of __________ [decedent name], who passed away in _________ [month and year of death].

Firstly, I would like to offer our condolences for your loss. I understand that you may be in a time of grief and this topic may bring up difficult memories, so I just wanted to check in – is this something that you feel comfortable discussing right now? If yes, are you in a place or can you move to a place where you have privacy to talk?

*[If no:]*

Ask if there is another time that they would prefer to be contacted at/that may be better for them.

*[If yes:]*

Okay great; thank you so much. We understand this can be difficult to talk about, so I really appreciate you taking the time to speak with me today. Please know that you can change your mind, we can reschedule at a time that works for you or remove you from our call list.

May I first share a little information about this project?

*[If yes:]*

We at San Francisco Department of Public Health are conducting this project to learn more about how we can prevent premature deaths. We are reaching out to individuals who know someone who passed away prematurely to interview them about many different aspects of their life. We have conducted over 100 interviews thus far. By conducting these interviews, we hope to better understand what may have led to their premature passing. The information people share is valuable and may help inform ways we can possibly prevent premature deaths.

Folks who are eligible and participate in our project complete a one-time, 1-2 hour interview either by phone, at our office here in San Francisco, or by Zoom. A $50 stipend is provided for those eligible to participate and who complete the survey.

What questions do you have about this project? Is this something you think you’d be willing to take part in?

*[If no:]*

Okay, thank you. Would it be okay if I try calling you again at another in a month or so to see if you feel the same or have changed your mind?

If no, we can remove from our call list if you like.

*[If they want to be removed from the list:]*

Okay, thank you for your time. We will take you off our call list. Is there anyone else that you would suggest I contact regarding this study who knew [decedent’s name]?

If you change your mind about participating and would like to know more about the study or think of anyone else who may want to hear about the study, please feel free to call me anytime at this number.

*[If yes:]*

*If willing to participate: Thank you for being willing to participate. The first step is for me to ask a few questions to see if you are eligible to participate.* ***Go through eligibility questions.***

*[If ineligible:]*

Unfortunately, it looks like based on your responses you are not eligible to participate in this study, but I thank you for taking the time to speak with me. Is there anyone else that you would suggest I contact regarding this study who knew [decedent’s name]?

*[If eligible:]*

Thank you very much for answering those questions. You are eligible to participate.May I share a bit more about the specifics of the study?

The objective of the study is to learn more about premature deaths, specifically related to the use of stimulants, such as methamphetamine and cocaine. Unfortunately, at this point, not much is known about why stimulant-related deaths happen. By interviewing people like you, who knew someone whose death was determined to be related to stimulant use, we hope to develop an intervention to help prevent future stimulant-related deaths.

Do you have any questions about what I just shared?

*During the survey we will go over topics within [decedent name]’s life such as their childhood and life experiences, medical information, personality, substance use history, suicidality, social networks, neighborhood and living conditions.* You may not have answers to all our questions and that is understandable. What you do share will be very helpful to us for creating future projects.

Are you comfortable in scheduling a day and time for this survey?

*[If no:]*

Okay, thank you for your time. We will take you off our call list. Is there anyone else that you would suggest I contact regarding this study who knew [decedent’s name]?

If you change your mind about participating and would like to know more about the study or think of anyone else who may want to hear about the study, please feel free to call me anytime at this number.

*[If yes:]*

Great! Thank you so much.

***Schedule interview, and if the interview is by phone or Zoom, send out info sheet and response scales by email or mail.***

# Stimulant Overdose/LASSO Study Informant Survey/Guide v1.5, March 15, 2023

***Instructions to interviewer:*** *Throughout this survey and interview guide, there are places where you may choose to modify the questions to be responsive to the informant. For example, there will be places throughout the survey where you will see “they / [insert decedent’s name]” or “their / [insert decedent’s name]” and based on the flow of the conversation, understandability of the questions, and the comfortability of the participant, you should choose to use “they” or “their” (or other appropriate pronoun) or the decedent’s first name.*

*The open-ended questions are a guide for the interviewer. Depending on the conversation and other survey responses, the interviewer may skip a question, ask questions in a different order, or ask a question that is responsive to the conversation that may not be included in this guide. At all times the interviewer will be responsive to what the participant shares and any questions that may arise. The prompts are included to help the interviewer narrow a question or ask it in a different way to help understandability or elicit responses.*

***Programming note:*** *All questions in “informant demographics” will be programmed with a “decline to answer” option. All other questions in the survey will have a “don’t know,” “decline to answer,” and “not asked” option.*

# Introduction

***Read aloud to the participant:*** *Thank you again for participating in this interview today. I appreciate you having this discussion with me, which at times might be difficult.*

*As we discussed, the interview will last approximately 1-2 hours. We will take a 10-minute break in the middle of the interview, and if you need additional breaks, please just let me know and we can take them whenever you'd like to.*

*I will ask some questions that have a specific response, such as a "yes" or "no" response, and other questions that are more open-ended. Some questions may feel repetitive. We don't expect you to know the answer to every question, and if you need any clarification about what is being asked - let me know and I can rephrase the question. If you have other questions that come up, including about how the coroner recorded [insert decedent's name]'s cause of death, I can answer them at the end of the interview. Please let me know if you need to take a break, want to skip a question, or stop the interview at any time.*

1. Decedent’s OCME ID Number:

1. Date of interview:

1. Interviewer’s initials:

1. Decedent’s first name: ____________ *(to be completed by survey administrator*)

1. Decedent ID:

1. Informant ID:

1. When did you first meet them / *[insert decedent’s name]*?

____________DD/MM/YYYY

1. When did you last speak with them / *[insert decedent’s name]*?

____________DD/MM/YYYY

1. Did the informant last speak with the decedent within 30 days of their death, or more than 30 days before their death? *(to be completed by survey administrator based off #1 and #8)* Met and last spoke with the decedent during the month before they died Met the decedent more than a month before they died, and spoke to them within a month of their death

Met the decedent more than a month before they died, and spoke to them last prior to a month before their death

1. What was your relationship to them / *[insert decedent’s name]*?

Family

Friend

Significant other/intimate partner

Neighbor

Service provider

Acquaintance

Other, specify:

1. *[If #10 = Family]* Please specify the familial relationship.

Mother

Father

Sister

Brother

Daughter

Son

Uncle

Aunt

Nephew

Niece

Other, specify:

12. *[If #10 = Service provider]* Please specify what type of service provider.

Clinician

Case manager

Counselor

Harm reduction services provider Residential staff (e.g., SRO manager) Other, specify:

**Demographics**

# Informant Demographics

1. What is your age? ________ years

1. What is your race?

African American or Black

Asian American

Native American, American Indian, or Alaskan Native

Native Hawaiian/Other Pacific Islander

White, Caucasian, or European American

Other (specify)

1. What is your ethnicity?

Non-Latino/Non-Hispanic

Latino/Hispanic

1. What is your gender? Genderqueer

Gender Variant, Gender Non-conforming

Man

Transgender Man/Trans Man

Transgender Woman/Trans Woman

Woman

Additional category, please specify: _________________

1. What is your assigned sex at birth?

Female

Male

Intersex

# Decedent Demographic Characteristics

1. What was their/ *[insert decedent’s name]* gender?

Genderqueer

Gender Variant, Gender Non-conforming

Man

Transgender Man/Trans Man

Transgender Woman/Trans Woman

Woman

Additional category, please specify: _________________

1. What is the highest level of schooling that they / *[insert decedent’s name]* completed?

Less than 8th grade

Some high school

High school graduate/GED

Trade/technical/vocational school

Some college

College graduate

Graduate degree

1. Which of these is closest to their / *[insert decedent’s name]* individual yearly income before taxes?

$0

$1 to $9,999

$10,000 to $24,999

$25,000 to $49,999

$50,000 to $74,999

$75,000 to $99,999

$100,000 to $149,999

$150,000 or more

1. Could you please describe your relationship to them / [*insert decedent’s name*]?

*Prompts:*

*Would you consider it a close relationship?*

*How often did you see them (annually, monthly, more regularly)?*

*Were there activities that you did together? If so, what were they?*

# Neighborhood and Housing

22. What neighborhood did they / *[insert decedent’s name]* most often stay in?

Bayview

Bernal Heights

Castro/Upper Market

Chinatown

Crocker-Amazon

Diamond Heights/Glen Park

Downtown/Civic Center

Excelsior

Financial District

Golden Gate Park

Haight Ashbury

Inner Richmond

Inner Sunset

Lakeshore

Marina

Mission

Mission Bay

Nob Hill

Noe Valley

North Beach

Oceanview

Outer Mission

Outer Richmond

Outer Sunset

Pacific Heights

Parkside

Potrero Hill

Presidio

Presidio Heights

Russian Hill

Sea Cliff

South of Market

Treasure Island YBI

Twin Peaks

Visitacion Valley

West of Twin Peaks

Western Addition

1. I’d like to know about their housing situation at the time they died. Where were they / *[insert decedent’s name]* living? For example, a house or apartment, single room occupancy, in a vehicle, or on the street.

Their home or apartment

My home or apartment

Someone else’s home or apartment

Abandoned building

Hotel room/Single Room Occupancy (SRO)

On the street, including in a tent or in a public space

Car or van

Shelter/navigation center

Residential treatment/inpatient psychiatric program

Other, specify

1. Please describe their / [*insert decedent’s name*]’s neighborhood and living environment.

*Prompts*

*Were their aspects of their neighborhood that they liked? Disliked?*

*Were their aspects of their living environment that they liked? Disliked?*

# Life Events and Adverse Childhood Experiences

*In the next two sets of questions, I am going to ask you about events and experiences that they / [insert decedent’s name] may have had or not had during their lifetime. I am first going to ask some questions in the year before they died and then I am going to ask about experiences they may have had when they were younger. I understand you may not know the answer to some of these questions. If you do not know an answer, or if you do not want to answer a question, please let us know.*

# The List of Threatening Events (LTE)

*In the next questions, several unpleasant events are listed. Please let us know if they experienced these events in the year prior to their death.*

1. They suffered a serious illness, injury, or an assault.

Yes

No

1. A serious illness, injury, or assault happened to one of their close relatives. Yes

No

1. Their parent, child, or spouse died.

Yes

No

1. A close family friend or another relative of theirs (aunt, cousin, grandparent) died. Yes

No

1. They had a separation due to marital difficulties.

Yes

No

1. They broke off a steady relationship. Yes

No

1. They had a serious problem with a close friend, neighbor, or relative. Yes

No

1. They became unemployed or they were seeking work unsuccessfully for more than one month. Yes

No

1. They were fired from a job.

Yes

No

1. They had a major financial crisis.

Yes

No

1. They had problems with the police and a court appearance.

Yes

No

1. Something they valued was lost or stolen.

Yes

No

1. They relapsed to substance use, or were not using drugs for a period of time and then started using them again.

Yes

No

1. They had a problem with child protective services.

Yes

No

1. They spent time in jail or prison.

Yes

No

1. They lost their housing.

Yes

No

1. How did experiencing this event/these events in the year before their / [insert decedent’s name] death impact them? Do you think this event/these events impacted their substance use? If so, how?

*Prompts: Were they experiencing more hardship than usual during the year before their death?*

Now, I'd like to ask you some questions about events that happened during their / *[insert decedent’s name]*’s childhood. All of these next few questions refer to the time period before they / *[insert decedent’s name]* were 18 years of age.

1. Did they live with anyone who was depressed, mentally ill, or suicidal?

Yes

No

1. Did they live with anyone who was a problem drinker or alcoholic?

Yes

No

1. Did they live with anyone who used illegal drugs or who abused prescription medications? Yes

No

1. Did they live with anyone who served time or was sentenced to serve time in prison, jail, or other correctional facility? Yes

No

1. Were their / *[insert decedent’s name]* parents separated or divorced?

Yes

No

Parents not married

1. How often did their parents or adults in their home ever slap, hit, kick, punch, or beat each other up?

Never

Once

More than once

1. Not including spanking, (before age 18), how often did a parent or adult in their home ever hit, beat, kick, or physically hurt them in any way?

Never

Once

More than once

1. How often did a parent or adult in their home ever swear at them, insult them, or put them down?

Never

Once

More than once

1. How often did anyone at least 5 years older than them or an adult, ever touch them sexually? Never

Once

More than once

1. How often did anyone at least 5 years older than them or an adult, try to make them touch the older person or adult sexually?

Never

Once

More than once

1. How often did anyone at least 5 years older than them or an adult, force them to have sex? Never

Once

More than once

1. Were they / *[insert decedent’s name] ever in foster care?*

1. How did experiencing this event/these events during their/ [insert decedent’s name] childhood impact them? Do you think this event/these events impacted their substance use? If so, how?

**Cardiovascular Health and Family History**

# Cardiovascular Review of Systems

*The following few questions are about [insert decedent’s name]’s cardiovascular health*

1. Did they / *[insert decedent’s name]* have any heart or cardiovascular problems?

Yes

No

*For the next few questions, we are interested in their cardiovascular health during the 30 days before their death [or year before their death if #9 = Met the decedent more than a month before they died, and spoke to them last prior to a month before their death].*

1. Did they / *[insert decedent’s name]* have chest pain or pressure?

Yes

No

1. *[If #56 = Yes]* Did they / *[insert decedent’s name]* have chest pain or pressure when they were using drugs?

Yes

No

1. Did they / *[insert decedent’s name]* have shortness of breath?

Yes

No

1. *[If #58 = Yes]* Did they / *[insert decedent’s name]* have shortness of breath only when they were walking or exercising, or also when they were at rest?

Only when walking or exercising

Both when walking or exercising and at rest

1. *[If #58 = Yes]* Did they / *[insert decedent’s name]* have shortness of breath when they were using drugs?

Yes

No

1. Did they / *[insert decedent’s name]* have calf or leg pain or cramps when they walked?

Yes

No

1. Did they / *[insert decedent’s name]* have leg swelling?

Yes

No

1. Did they / *[insert decedent’s name]* pass out / lose consciousness for a reason other than an opioid overdose? This may have involved other drugs or some other medical reason.

Yes

No

1. *[If #63 = Yes]* Did they / *[insert decedent’s name]* pass out / lose consciousness when they were using drugs?

Yes

No

1. Did they / *[insert decedent’s name]* have a rapid heartbeat or palpitations?

Yes

No

1. *[If #65 = Yes]* Did they / *[insert decedent’s name]* have a rapid heartbeat or palpitations when they were using drugs?

Yes

No

# Family History

*The following three questions are about [insert decedent’s name]’s family medical history. When we refer to an “immediate family member” we mean their parent, grandparent, or siblings.*

1. Did they / *[insert decedent’s name]* have any immediate family members who died suddenly because of a heart problem or without a clear cause of death?

Yes

No

1. *[If #67 = Yes]* Which family member(s) was it? Mark all that apply.

Mother

Father

Grandmother

Grandfather

Sister (including half-sisters)

Brother (including half-brothers)

1. Did they/ *[insert decedent’s name]* have any immediate family members who would pass out / lose consciousness or collapse?

Yes

No

1. *[If #69 = Yes]* Which family member(s) was it? Mark all that apply.

Mother

Father

Grandmother

Grandfather

Sister (including half-sisters)

Brother (including half-brothers)

1. Did they / *[insert decedent’s name]* haveany immediate family members who had multiple miscarriages?

Yes

No

1. *[If #71 = Yes]* Which family member(s) was it? Mark all that apply.

Self

Mother

Grandmother

Sister (including half-sisters)

Brother (including half-brothers)

1. Did they / [*insert decedent’s name*] ever share concerns with you about their physical health that they were not already getting medical care for? If so, please describe.

1. We are interested in how an undiagnosed heart condition could be complicated by stimulant use. Were there any other heart problems that they told you about, which we haven’t talked about? Including any symptoms or conditions that they knew about and weren’t getting help for?

1. How did their / *[insert decedent’s name]* medical conditions influence their substance use? For example, did they try to reduce their use or use in ways they thought were safer? Or did they do the opposite?

**Substance Use**

# Lifetime, Past Year, and Past 30-Day Substance Use

The next several questions will ask about their / *[insert decedent’s name]*’s use of drugs during:

*(If #9 = “Met and last spoke with the decedent during the month before they died”):* during the 30 days before their death.

*(If #9= “Met the decedent more than a month before they died and spoke to them within a month of their death”):* their lifetime, the year before their death, and the 30 days before their death.

*(If #9 = “Met the decedent more than a month before they died and spoke to them last prior to a month before their death”):* their lifetime and the year before their death.

76. *[If #9≠ “Met and last spoke with the decedent during the month before they died]* In their lifetime, which of the following drugs are you aware they / *[insert decedent’s name]* had used? Please check all that apply.

Marijuana or hashish

Poppers or inhaled nitrates

Powder cocaine

Crack cocaine

Methamphetamine or speed

Heroin

Medicinal opioids, such as oxycodone, hydrocodone, morphine, and codeine Fentanyl

Goofball (heroin/fentanyl and methamphetamine together)

Speedball (heroin/fentanyl and cocaine together)

GHB or GBL, also called G

Ecstasy or Molly

Ketamine, also called Special K

Hallucinogens, such as PCP, Angel Dust, Acid, LSD, or Mushrooms

Benzodiazepines, such as Valium, Ativan, and Xanax

Alcohol

Tobacco smoking/vaping

Something else (specify)

None

77. *[If #9≠ “Met and last spoke with the decedent during the month before they died]* In the year before their death, which of the following drugs are you aware they / *[insert decedent’s name]* had used? *[Show each of the drugs selected in #76]* Please check all that apply.

Marijuana or hashish

Poppers or inhaled nitrates

Powder cocaine

Crack cocaine

Methamphetamine or speed

Heroin

Medicinal opioids, such as oxycodone, hydrocodone, morphine, and codeine Fentanyl

Goofball (heroin/fentanyl and methamphetamine together)

Speedball (heroin/fentanyl and cocaine together)

GHB or GBL, also called G

Ecstasy or Molly

Ketamine, also called Special K

Hallucinogens, such as PCP, Angel Dust, Acid, LSD, or Mushrooms Benzodiazepines, such as Valium, Ativan, and Xanax

Alcohol

Tobacco smoking/vaping

Something else (specify)

None

1. *[If #77= Fentanyl]* Had they used fentanyl that was not prescribed in the year before their death? Yes

No

1. *[If #78 = Yes]* How often did they / *[insert decedent’s name]* use fentanyl in the year before their death?

Every day

At least weekly, but not every day

At least monthly, but not every week

Less than monthly

1. *[If #78 = Yes]* How often when they / *[insert decedent’s name]* used fentanyl in the year before their death, were they intending to use fentanyl?

Always - every time they used fentanyl, they were intending to use fentanyl Often

Sometimes

Rarely

Never - every time they used fentanyl, they were NOT intending to use it

1. *[If #78 = Yes]* How did they / *[insert decedent’s name]* use fentanyl in the year before their death? (Please select all that apply)

Injected

Skin popped it

Inserted it rectally (booty bumped)

Snorted it

Smoked it

Eaten it, ingested it orally

Other (specify)

1. *[If #77 = Methamphetamine or speed]* How often did they / *[insert decedent’s name]* use methamphetamine in the year before their death?

Every day

At least weekly, but not every day

At least monthly, but not every week

Less than monthly

1. *[If #77 = Methamphetamine or speed]* How did they / *[insert decedent’s name]* use methamphetamine in the year before their death? (Please select all that apply)

Injected it

Inserted it rectally (booty bumped)

Snorted it

Smoked it / chased it

Swallowed it, ingested it orally

Other (specify)

1. *[If #77 = Powder cocaine or Crack cocaine]* How often did they / *[insert decedent’s name]* use cocaine in the year before their death?

Every day

At least weekly, but not every day

At least monthly, but not every week

Less than monthly

1. *[If #77 = Powder cocaine or Crack cocaine]* How did they / *[insert decedent’s name]* use cocaine in the year before their death? (Please select all that apply)

Injected it

Inserted it rectally (booty bumped)

Snorted it

Smoked it /chased it

Swallowed it, ingested it orally

Other (specify)

1. *[If #9 ≠ Met the decedent more than a month before they died and spoke to them last prior to a month before their death]:* Which of the following drugs are you aware they / *[insert decedent’s name]* had used in the 30 days before their death?

Marijuana or hashish

Poppers or inhaled nitrates

Powder cocaine

Crack cocaine

Methamphetamine or speed

Heroin

Medicinal opioids, such as oxycodone, hydrocodone, morphine, and codeine Fentanyl

Goofball (heroin/fentanyl and methamphetamine together)

Speedball (heroin/fentanyl and cocaine together)

GHB or GBL, also called G

Ecstasy or Molly

Ketamine, also called Special K

Hallucinogens, such as PCP, Angel Dust, Acid, LSD, or Mushrooms Benzodiazepines, such as Valium, Ativan, and Xanax

Alcohol

Tobacco smoking/vaping

Something else (specify)

None

1. *[If #86= Fentanyl]* Had they / *[insert decedent’s name]* used fentanyl that was not prescribed in the 30 days before their death?

Yes

No

1. *[If #87 = Yes]* How often did they / *[insert decedent’s name]* use fentanyl in the 30 days before their death?

Every day

More than once a week, but not every day

Every week

Once in those 30 days

1. *[If #87 = Yes]* How often when they / *[insert decedent’s name]* used fentanyl in the 30 days before their death, were they intending to use fentanyl?

Always - every time they used fentanyl, they were intending to use fentanyl Often

Sometimes

Rarely

Never - every time they used fentanyl, they were NOT intending to use it

1. *[If #87 = Yes]* How did they/ *[insert decedent’s name]* use fentanyl in the 30 days before their death? (Please select all that apply)

Injected it into vein

Skin popped it

Inserted it rectally (booty bumped)

Snorted it

Smoked it

Eaten it, ingested it orally

Other (specify)

1. *[If #86 =Methamphetamine or speed]* How often did they / *[insert decedent’s name]* use methamphetamine in the 30 days before their death? Every day

More than once a week, but not every day

Every week

Once in those 30 days

1. *[If #86 =Methamphetamine or speed]* How did they / *[insert decedent’s name]* use methamphetamine in the 30 days before their death? (Please select all that apply)

Injected it

Inserted it rectally (booty bumped)

Snorted it

Smoked it / chased it

Swallowed it, ingested it orally

Other (specify)

1. *[If #86 = Powder cocaine or crack cocaine]* How often did they / *[insert decedent’s name]* use cocaine in the 30 days before their death?

Every day

More than once a week, but not every day

Every week

Once in those 30 days

1. *[If #86 = Powder cocaine or crack cocaine]* How did they / *[insert decedent’s name]* use cocaine in the 30 days before their death? (Please select all that apply)

Injected it

Inserted it rectally (booty bumped)

Snorted it

Smoked it /chased it

Swallowed it, ingested it orally

Other (specify)

1. *[If #76, #77 or #86 =Methamphetamine or speed]* Approximately how old were they/ [insert decedent’s name] when they first used methamphetamine or speed?

_______ years old

1. *[If #76, #77 or #86 =Powder cocaine or crack cocaine]* Approximately how old were they/ [insert decedent’s name] when they first used powder cocaine or crack cocaine?

_______ years old

# Severity of Dependence on Substances

*[If #78 = Yes OR #87 = Yes]* The next few questions are going to ask about how they / *[insert decedent’s name]* felt about their fentanyl use in the year before they died.

1. *[If #78 = Yes OR #87 = Yes]* Did they / *[insert decedent’s name]* think their use of fentanyl use was out of control?

Never / almost never

Sometimes

Often

Always /nearly always

1. *[If #7877 = Yes OR #87 = Yes]* Did the prospect of missing a hit (line, dose) of fentanyl make them / *[insert decedent’s name]* very anxious or worried?

Never / almost never

Sometimes

Often

Always / nearly always

1. *[If #78 = Yes OR #87 = Yes]* Did they worry about their / *[insert decedent’s name]* use of fentanyl?

Never / almost never

Sometimes

Often

Always / nearly always

1. *[If #78 = Yes OR #87 = Yes]* Did they wish they / *[insert decedent’s name]* could stop using fentanyl?

Never / almost never

Sometimes

Often

Always / nearly always

1. *[If #78 = Yes OR #87 = Yes]* How difficult would they / *[insert decedent’s name]* have found it to stop or go without fentanyl?

Not difficult

Quite difficult

Very difficult

Impossible

*[If #77 = Methamphetamine or speed OR #86 = Methamphetamine or speed]* The next few questions are going to ask about how they / *[insert decedent’s name]* felt about their methamphetamine or speed use in the year before they died.

1. *[If #77 = Methamphetamine or speed OR #86 = Methamphetamine or speed]* Did they / *[insert decedent’s name]* think their use of methamphetamine was out of control?

Never / almost never

Sometimes

Often

Always /nearly always

1. *[If #77 = Methamphetamine or speed OR #86 = Methamphetamine or speed]* Did the prospect of missing a hit (line, dose) of methamphetamine make them / *[insert decedent’s name]* very anxious or worried?

Never / almost never

Sometimes

Often

Always / nearly always

1. *[If #77 = Methamphetamine or speed OR #86 = Methamphetamine or speed]* Did they / *[insert decedent’s name]* worry about their use of methamphetamine?

Never / almost never

Sometimes

Often

Always / nearly always

1. *[If #77 = Methamphetamine or speed OR #86 = Methamphetamine or speed]* Did they / *[insert decedent’s name]* wish they could stop using methamphetamine?

Never / almost never

Sometimes

Often

Always / nearly always

1. *[If #77 = Methamphetamine or speed OR #86 = Methamphetamine or speed]* How difficult would they / *[insert decedent’s name]* have found it to stop or go without methamphetamine? Not difficult

Quite difficult

Very difficult

Impossible

*[If #77 = Powder cocaine or crack cocaine OR #86 = Powder cocaine or crack cocaine]* The next few questions are going to ask about how they / *[insert decedent’s name]* felt about their powder cocaine or crack cocaine use in the year before they died.

1. *[If #77 = Powder cocaine or crack cocaine OR #86 = Powder cocaine or crack cocaine]* Did they / *[insert decedent’s name]* think their use of cocaine was out of control?

Never / almost never

Sometimes

Often

Always /nearly always

1. *[If #77 = Powder cocaine or crack cocaine OR #86 = Powder cocaine or crack cocaine]* Did the prospect of missing a hit (line, dose) of cocaine make them / *[insert decedent’s name]* very anxious or worried?

Never / almost never

Sometimes

Often

Always / nearly always

1. *[If #77 = Powder cocaine or crack cocaine OR #86 = Powder cocaine or crack cocaine]* Did they / *[insert decedent’s name]* worry about their use of cocaine?

Never / almost never

Sometimes

Often

Always / nearly always

1. *[If #77 = Powder cocaine or crack cocaine OR #86 = Powder cocaine or crack cocaine]* Did they / *[insert decedent’s name]* wish they could stop using cocaine?

Never / almost never

Sometimes

Often

Always / nearly always

1. *[If #77 = Powder cocaine or crack cocaine OR #86 = Powder cocaine or crack cocaine]* How difficult would they / *[insert decedent’s name]* have found it to stop or go without cocaine? Not difficult

Quite difficult

Very difficult

Impossible

# Substance Use Qualitative Questions

1. *Is there anything else you would like to share about their / [insert decedent’s name]’s substance use in the year [If #9 ≠ Met the decedent more than a month before they died and spoke to them last prior to a month before their death add “or 30 days”] before their death?*

*Prompts*

*For example, if you were present when they used drugs, could you describe their patterns of use at all?*

*Were they using more or less, more or less frequently?*

*Were they using in more chaotic ways?*

*What types of problems, if any, did they tend to have when they used?*

1. *Were you or they* / *[insert decedent’s name]* *concerned about any aspects of their substance use? If so, could you please describe what aspects of their substance use were concerning?*

1. How did their / *[insert decedent’s name]’s* neighborhood or environment impact their substance use?

*Prompts*

*Were there aspects of their neighborhood or environment that made it harder or easier for them to reduce or manage their substance use?*

# Fatal Overdose Experience/ Drug-Related Emergency

*Now we are going to talk about overdose experiences. We realize this may bring up a lot of feelings, so please let us know if you need to take a break. We are also going to ask you about the events surrounding [insert decedent’s name/their] death. As we mentioned at the beginning of the interview, if you have additional questions that we can answer about those events, we would be happy to talk more at the end of the survey. Thank you.*

1. Are you aware of how they/ *[insert decedent’s name]* died?

Yes / Has some information

No

1. *[If #115 = Yes]* To your knowledge, how did they / *[insert decedent’s name]* die? Please check all that apply.

Opioid overdose

Methamphetamine overdose

Cocaine overdose

Drug overdose (not otherwise specified)

Heart attack

Stroke

Infection

Other, please specify

1. *[*If #*116 = Opioid, methamphetamine, or cocaine overdose]* Where did that overdose occur?

Their home or apartment

My home or apartment

Someone else’s home or apartment

In a hospital

Abandoned building

Hotel room/Single Room Occupancy (SRO)

On the street, including in a tent or in a public space

Car or van

Other, specify

1. [If #*116 = Opioid, methamphetamine, or cocaine overdose*] What was done in response to the overdose? Please select all that apply.

Naloxone was administered

CPR

Someone called 911

They were taken to directly hospital Other, specify

Nothing

1. *[If #116 = Opioid overdose]* Approximately how many times would you say they / *[insert decedent’s name]* had an opioid overdose in the year before they died?

___________ # of overdoses

1. Were you present around the time when they / *[insert decedent’s name]* died?

Yes

No

1. *[If #120 = Yes]* Was this the whole time, before they / *[insert decedent’s name]* died, or only after they / *[insert decedent’s name]* had passed?

The whole time (before and after death)

Before they died

After they had died

1. *[If #120 = Yes]* Were they / *[insert decedent’s name]* using drugs before they died?

Yes

No

1. *[If # 122= Yes]* What drugs were they / *[insert decedent’s name]* using before they died? Please check all that apply.

Marijuana or hashish

Poppers or inhaled nitrates

Powder cocaine

Crack cocaine

Methamphetamine or speed

Heroin

Medicinal opioids, such as oxycodone, hydrocodone, morphine, and codeine Fentanyl

Goofball (heroin/fentanyl and methamphetamine together)

Speedball (heroin/fentanyl and cocaine together)

GHB or GBL, also called G

Ecstasy or Molly

Ketamine, also called Special K

Hallucinogens, such as PCP, Angel Dust, Acid, LSD, or Mushrooms

Benzodiazepines, such as Valium, Ativan, and Xanax

Alcohol

None

Something else (specify)

1. *[If #123 = Fentanyl]* To your knowledge, did they / *[insert decedent’s name]* mean to use fentanyl when they died?

Yes

No

1. [*If* #120 *= Yes] Is there anything else you’d like to share with me about this experience? Was there anything else that either happened before, during, or after their death that stands out to you?*

# Previous Non-Fatal Opioid Overdose Experiences/ Drug-Related Emergencies

1. *[If #115 = No OR #116 ≠ Opioid overdose]* In their lifetime, did they / *[insert decedent’s name]* ever have an opioid overdose?

Yes

No

1. *[If #126>Yes]* Approximately how many times would you say they / *[insert decedent’s name]* had an opioid overdose in the year before they died?

___________ # of overdoses

1. *[If #127>0]* When was the last time they / *[insert decedent’s name]* had an opioid overdose that you are aware of in the year before they died?

__________ MM/DD/YYYY

1. *[If #127>0]* Where did that overdose occur?

Their home or apartment

My home or apartment

Someone else’s home or apartment

Abandoned building

Hotel room/Single Room Occupancy (SRO)

On the street

Car or van

Other, specify

1. *[If #127>0]* Were you present during that overdose?

Yes

No

1. *[If #127>0]* Which drugs were they using when that overdose occurred?

Marijuana or hashish

Poppers or inhaled nitrates

Powder cocaine

Crack cocaine

Methamphetamine or speed

Heroin

Medicinal opioids, such as oxycodone, hydrocodone, morphine, and codeine Fentanyl

Goofball (heroin/fentanyl and methamphetamine together)

Speedball (heroin/fentanyl and cocaine together)

GHB or GBL, also called G

Ecstasy or Molly

Ketamine, also called Special K

Hallucinogens, such as PCP, Angel Dust, Acid, LSD, or Mushrooms

Benzodiazepines, such as Valium, Ativan, and Xanax

Alcohol

Something else (specify)

1. *[If #131 = Fentanyl]* To your knowledge, did they / [*insert decedent’s name*] mean to use fentanyl when they last overdosed?

Yes

No

1. *[If #127>0]* What was done in response to that overdose? (Please select all that apply.) Naloxone was administered

CPR

Someone called 911

They were taken to directly hospital Other, specify

Nothing

1. *[If #127>0]* Is there anything else you’d like to share with me about their experiences of opioid overdose?

1. *[If #76 OR #77 OR #86 =Heroin, Medicinal opioids, or Fentanyl]* Were they / *[insert decedent’s name]* concerned about having an opioid overdose? If yes, what were some things they did to prevent opioid overdose, if anything?

*We are also going to ask you about taking too many stimulants (i.e., methamphetamine or cocaine), or “overamping,” which is sometimes called a stimulant overdose. This can involve severe physical or mental health symptoms that someone experiences as a result of using stimulants. Physical health events might be a heart attack, stroke, seizure, or intense overheating. Mental health events might be* ***extreme*** *panic, paranoia, anxiety or agitation, hallucinations, or psychosis. With these definitions in mind...*

1. Did they / *[insert decedent’s name]* ever tell you that they felt like they were having a heart attack, stroke, or seizure while using methamphetamine or cocaine?

Yes

No

1. Did they / *[insert decedent’s name]* ever tell you that they felt like they were losing their mind, manic, or psychotic while using methamphetamine or cocaine?

Yes

No

1. *[If #136 or #137 = Yes]* Approximately how many times would you say they / *[insert decedent’s name]* overamped, with any of the symptoms we’ve noted, in the year before they died?

___________ # of overamps

1. [If #138>0] What symptoms did they / *[insert decedent’s name]* tend to have when they overamped during the year before they died? Please check all that apply.

Racing heart

Skipped heart beats

Chest pain

Shortness of breath

Sweating

Seizure

Intense overheating

Panic attack

Agitation / can’t stop moving

Anxiety

Paranoia

Hallucinations

Psychosis

Other ___

1. *[If #138>0 AND #116 ≠ Methamphetamine overdose or Cocaine overdose]* When was the last time they / *[insert decedent’s name]* overamped on stimulants that you are aware of in the year before they died?

__________ MM/DD/YYYY

1. *[If #138>0 AND #116 ≠ Methamphetamine overdose or Cocaine overdose]* Can you please describe how they / *[insert decedent’s name]* were using stimulants the last time they overamped?

*Prompts*

*How many hours or days had they been using stimulants?*

*Had they been using stimulants differently before overamping? A different route of administration? Different patterns of use (e.g., binge use)?*

1. *[If #138>0 AND #116 ≠ Methamphetamine overdose or Cocaine overdose]* What did they / *[insert decedent’s name]* do, or did someone else do, in response to that overamping event? *Prompts*

*Did they take any medication?*

*Were emergency services or 911 called?*

*Did someone try to relax them?*

1. *[If #76 OR #77 OR #86 =Methamphetamine or speed, Powder cocaine, or Crack cocaine]* Were they / *[insert decedent’s name]* concerned about overamping? If yes, what were some things they / *[insert decedent’s name]* did to prevent overamping, if anything?

# Substance Use Harm Reduction Services

1. In their lifetime, did they / *[insert decedent’s name]* ever receive any type of professional help for their use of alcohol or drugs? By help we mean a detox or rehabilitation center, methadone, buprenorphine or other medication, or any other type of program or meeting that helped them reduce or stop their alcohol or drug use.

No

Yes, for drugs only

Yes, for alcohol only

Yes, for both drugs and alcohol

1. *[If #144 ≠ No]* In their lifetime, what type of treatment did they / *[insert decedent’s name]* receive for their alcohol or drug use?

Residential drug or alcohol treatment

Outpatient substance use counseling

Methadone maintenance treatment

Buprenorphine (suboxone) treatment

Inpatient detoxification

Vivitrol (naltrexone) treatment

Sober House

AA/NA or another group

Mandated (court-ordered) treatment

Contingency management

Other, specify

1. *[If #144 ≠ No]* Did they / *[insert decedent’s name]* receive any of these treatments in the year before they died?

Yes

No

1. *[If #146 = Yes]* Which treatments did they / *[insert decedent’s name]* receive in the year before they died? (Please select all that apply.)

Residential drug or alcohol treatment

Outpatient substance use counseling

Methadone maintenance treatment

Buprenorphine (suboxone) treatment

Inpatient detoxification

Vivitrol (naltrexone) treatment

Sober House

AA/NA or another group

Mandated (court-ordered) treatment

Contingency management

Other, specify

1. Did they / *[insert decedent’s name]* use a syringe services program/needle exchange in the year before they died?

Yes

No

1. *[If #148 = Yes]* How often did they / *[insert decedent’s name]* access services at a syringe services program/needle exchange in the year before they died?

Monthly or less

2 to 4 times a month

2 to 3 times a week

4 or more times a week

1. When they / *[insert decedent’s name]* were using substances, how often in the year before they died did they have their own Narcan/naloxone with them? *[Prompt if needed: Naloxone is a medication that can be used to reverse an opioid overdose. It is often injected into a muscle or sprayed up an overdose victim’s nose.]*

Never

Rarely

Sometimes

Often

Always

1. When they / *[insert decedent’s name]* were using substances, how often in the year before they died did they use a fentanyl test strip

Never

Rarely

Sometimes

Often

Always

1. *[If #151= Yes]* Did the results have no effect on their drug use, lead them / *[insert decedent’s name]* to avoid fentanyl, or were they seeking fentanyl?

The results had no effect on their drug use

The result led them to avoid fentanyl

They were seeking fentanyl

1. *[If #77 ≠ None AND #86 ≠ None]* What other strategies did they / *[insert decedent’s name]* use to try to keep themselves safe while using drugs in the year before they died?

1. *[If #77 ≠ None AND #86 ≠ None]* Are there any other ways they / *[insert decedent’s name]* managed or reduced their use in the year before they died that we haven’t discussed?

*Prompts*

*Transitioned to other drugs? If so, which ones?*

*Injected drugs less frequently?*

*Transitioned route of administration (e.g., from injecting to smoking)?*

# Impulsivity

*People are different in the ways they think and act in different situations. We are going to now ask a few questions to get a better understanding of how they / [insert decedent’s name] thought and acted in the 30 days prior to their death [or “year before their death” if #9 = Met the decedent more than a month before they died, and spoke to them last prior to a month before their death]. Please listen to the following statements and tell me if they acted that way never or rarely, sometimes, often, or almost always or always.*

1. They planned tasks carefully.

Rarely/Never

Sometimes

Often

Almost Always/Always

1. They did things without thinking.

Rarely/Never

Sometimes

Often

Almost Always/Always

1. They didn’t “pay attention.”

Rarely/Never

Sometimes

Often

Almost Always/Always

1. They were self-controlled.

Rarely/Never

Sometimes

Often

Almost Always/Always

1. They concentrated easily.

Rarely/Never

Sometimes

Often

Almost Always/Always

1. They were a careful thinker.

Rarely/Never

Sometimes

Often

Almost Always/Always

1. They said things without thinking.

Rarely/Never

Sometimes

Often

Almost Always/Always

1. They acted in the spur of the moment.

Rarely/Never

Sometimes

Often

Almost Always/Always

# Resilience

*The next few questions will assess resilience, or the ability that they / [insert decedent’s name] had to handle stress. We are going to read a series of statements about them / [insert decedent’s name]; please tell us if* ***you*** *strongly disagree, disagree, neither disagree or agree, agree, or strongly agree with each statement.*

1. They tended to bounce back quickly after hard times.

Strongly Disagree

Disagree

Neither disagree nor agree

Agree

Strongly Agree

1. They had a hard time making it through stressful events.

Strongly Disagree

Disagree

Neither disagree nor agree

Agree

Strongly Agree

1. It did not take a long time for them to recover from a stressful event.

Strongly Disagree

Disagree

Neither disagree nor agree

Agree

Strongly Agree

1. It was hard for them to snap back when something bad happened.

Strongly Disagree

Disagree

Neither disagree nor agree

Agree

Strongly Agree

1. They usually came through difficult times with little trouble.

Strongly Disagree

Disagree

Neither disagree nor agree

Agree

Strongly Agree

1. They tended to take a long time to get over setbacks in their life.

Strongly Disagree

Disagree

Neither disagree nor agree

Agree

Strongly Agree

# Mental and Emotional Health (Depression, Anxiety, Suicidality)

1. Did they/ *[insert decedent’s name]* name have any mental and/or emotional health disorders? Yes

No

1. *[If #169 = Yes]* Which mental and/or emotional health disorders did they/ *[insert decedent’s name]* have? Please select all that apply.

Depression

Anxiety or panic disorders or phobias

Obsessive-compulsive disorder

Bipolar disorder

Post-traumatic stress disorder

Schizophrenia

Other, specify:

*We are now going to ask some questions about their / [insert decedent’s name]’s mood during the 30 days before their death [or year before their death if #9 = Met the decedent more than a month before they died, and spoke to them last prior to a month before their death]. These questions will help us better understand not only their physical health when they died, but also their emotional health. We understand that these questions can be difficult to answer; please remember you do not have to answer any question you do not want to.*

# PHQ-8

We will first ask how often (not at all, several days, more than half the days, or nearly every day) they were bothered by the following:

1. Little interest or pleasure in doing things.

Not at all

Several days

More than half the days

Nearly every day

1. Feeling down, depressed, or hopeless.

Not at all

Several days

More than half the days

Nearly every day

1. Trouble falling or staying asleep, or sleeping too much.

Not at all

Several days

More than half the days

Nearly every day

1. Feeling tired or having little energy.

Not at all

Several days

More than half the days

Nearly every day

1. Poor appetite or overeating.

Not at all

Several days

More than half the days

Nearly every day

1. Feeling bad about themselves— or that they were a failure or had let themselves or their family down

Not at all

Several days

More than half the days

Nearly every day

1. Trouble concentrating on things, such as reading the newspaper or watching television.

Not at all

Several days

More than half the days

Nearly every day

1. Moving or speaking so slowly that other people could have noticed. Or the opposite – being so fidgety or restless that they had been moving a lot more than usual.

Not at all

Several days

More than half the days Nearly every day

# GAD-2

1. Feeling nervous, anxious or on edge

Not at all

Several days

More than half the days Nearly every day

1. Not being able to stop or control worrying

Not at all

Several days

More than half the days

Nearly every day

1. Did their / [*insert decedent’s name*]'s mood change at all in the 30 days prior to their death *[or year before their death if #9 = Met the decedent more than a month before they died, and spoke to them last prior to a month before their death]*?

1. Did they / [*insert decedent’s name*] ever share concerns with you about their emotional or psychiatric health that they were not already getting care for? If so, please describe.

1. Did any aspects of their emotional or mental health impact their substance use? If so, how?

# Columbia Suicide Severity Rating Scale

*We are going to ask just a few more questions about their emotional health and mood before they died.*

*The next few questions are asking about the 30 days before they died [or year before their death if #9 = Met the decedent more than a month before they died and spoke to them last prior to a month before their death].*

1. Did they / *[insert decedent’s name]* wish they were dead or wish they could go to sleep and not wake up?

Yes

No

1. Did they / *[insert decedent’s name]* have any thoughts of killing themselves?

Yes

No

1. *[If #185 = Yes]* Did they / *[insert decedent’s name]* think about how they might do it?

Yes

No

1. *[If #185 = Yes]* Did they / *[insert decedent’s name]* have these thoughts and have some intention of acting on them?

Yes

No

1. *[If #185 = Yes]* Did they / *[insert decedent’s name]* start to work out or had worked out the details of how they were going to kill themselves? Did they intend to carry out this plan?

Yes

No

*The next question is not just about the month or year before they died, but their lifetime.*

1. Did they / *[insert decedent’s name]* ever do anything, start to do anything, or prepare to do anything to end their life?

Yes

No

1. *[If #189 = Yes]* Were any of these in the 3 months before they / *[insert decedent’s name]* died?

Yes No

# Social Environment

*In our final three sections of the interview, we are going to ask you about the community and social environment around them / [insert decedents’ s name]. We will first ask you questions about the relationships they had and the groups they belonged to during the year before they died.*

# Short Social Capital Assessment Tool (SA-SCAT)

1. In the 12 months before they / *[insert decedent’s name]* died, were they an active member of any of the following types of groups in their community? Please check all that apply.

Work-related/ trade union

Community association/ co-op

Women’s group

Political group

Religious group Sports group Other group, specify:

1. In the 12 months before they / *[insert decedent’s name]* died, did they receive from the group any emotional help, economic help, or assistance in helping them know or do things?

Work-related/ trade union

Community association/ co-op

Women’s group

Political group

Religious group Sports group

Other group, specify:

1. In the 12 months before they / *[insert decedent’s name]* died, did they receive any help or support from any of the following, this can be any emotional help, economic help, or assistance in helping them know or do things?

Family

Neighbors

Friends who are not neighbors

Community leaders

Religious leaders

Politicians

Government officials/civil service

Charitable organizations/NGO

Other

1. In the 12 months before they / *[insert decedent’s name]* died, had they joined together with other community members to address a problem or common issue?

Yes

No

1. In the 12 months before they / *[insert decedent’s name]* died, had they talked with a local authority or governmental organization about problems in their community?

Yes

No

1. In general, did they / *[insert decedent’s name]* believe that the majority of people in their community could be trusted?

Yes

No

1. Did they / *[insert decedent’s name]* believe that the majority of people in their community generally get along with each other?

Yes

No

1. Did they / *[insert decedent’s name]* feel as though they were really a part of their community? Yes

No

1. Did they / *[insert decedent’s name]* think that the majority of people in their community would try to take advantage of them if they got the chance?

Yes

No

# Multidimensional Scale of Perceived Social Support

*For this final set of questions, we will read a series of statements of how they / [insert decedent’s name] may have felt about the people around them during the year before they died. Please let me know if they would strongly disagree, disagree, neither disagree or agree, mildly agree, or strongly agree with each statement.*

1. There was a special person who was around when they were in need.

Strongly Disagree

Mildly Disagree

Neither disagree nor agree

Mildly Agree

Strongly Agree

1. There was a special person with whom they could share their joys and sorrows.

Strongly Disagree

Mildly Disagree

Neither disagree nor agree

Mildly Agree

Strongly Agree

1. Their family really tried to help them.

Strongly Disagree

Mildly Disagree

Neither disagree nor agree

Mildly Agree

Strongly Agree

1. They got the emotional help and support they needed from their family.

Strongly Disagree

Mildly Disagree

Neither disagree nor agree

Mildly Agree

Strongly Agree

1. They had a special person who was a real source of comfort to them.

Strongly Disagree

Mildly Disagree

Neither disagree nor agree

Mildly Agree

Strongly Agree

1. Their friends really tried to help them.

Strongly Disagree

Mildly Disagree

Neither disagree nor agree

Mildly Agree

Strongly Agree

1. They could count on their friends when things went wrong.

Strongly Disagree

Mildly Disagree

Neither disagree nor agree

Mildly Agree

Strongly Agree

1. They could talk about their problems with their family.

Strongly Disagree

Mildly Disagree

Neither disagree nor agree

Mildly Agree

Strongly Agree

1. They had friends with whom they could share their joys and sorrows.

Strongly Disagree

Mildly Disagree

Neither disagree nor agree

Mildly Agree

Strongly Agree

1. There was a special person in their life who cared about their feelings.

Strongly Disagree

Mildly Disagree

Neither disagree nor agree

Mildly Agree

Strongly Agree

1. Their family was willing to help them make decisions.

Strongly Disagree

Mildly Disagree

Neither disagree nor agree

Mildly Agree

Strongly Agree

1. They could talk about their problems with their friends.

Strongly Disagree

Mildly Disagree

Neither disagree nor agree

Mildly Agree

Strongly Agree

1. Is there anything else you’d like to add about what type of relationships they / *[insert decedent’s name]’s* had with family, friends, neighbors, or others?

1. How did these / *[insert decedent’s name]’s* relationships with family, friends, or others impact their substance use?

*Prompts*

*Did they use substances with friends, family, or others?*

*Did these relationships make it easier or harder for them to reduce or manage their substance use?*

Thank you so much for your time today.

1. *How was this experience? Did anything come up for you during this interview that you’d like resources for?*

1. *Is there anything that came up that might be helpful/relevant to this research that we didn’t discuss?*

1. *Is there anyone else we should reach out to who might be interested in participating in an interview?*

1. *Can we contact you in the future to follow-up about other people who could participate in an interview?*
